# Depression and Heart Failure With Preserved Ejection Fraction: An Inflammatory Perspective

**DOI:** 10.1155/da/7169650

**Published:** 2026-10-25

**Authors:** Hongfan Chen, Nan Zhang, Yufan Feng, Mengxing Tao, Chi Geng

**Affiliations:** ^1^ Department of Cardiology The Second Affiliated Hospital of Soochow University Suzhou 215004 Jiangsu China suda.edu.cn; ^2^ Suzhou Medical College of Soochow University Suzhou 215123 Jiangsu China suda.edu.cn; ^3^ Department of Pathophysiology Chinese Academy of Medical Sciences School of Basic Medicine Institute of Basic Medicine Peking Union Medical College Beijing 100005 China pumc.edu.cn; ^4^ Department of Rehabilitation Medicine The Affiliated Suzhou Hospital of Nanjing Medical University Suzhou Municipal Hospital Gusu School Nanjing Medical University Suzhou 215002 Jiangsu China njmu.edu.cn

**Keywords:** cardiac remodeling, comorbidity, depression, heart failure with preserved ejection fraction, inflammation

## Abstract

Depression is a common comorbidity in patients with heart failure with preserved ejection fraction (HFpEF), suggesting potential epidemiological and pathophysiological connections between these two age‐related chronic diseases. Extensive clinical evidence demonstrates that depression is closely associated with both the incidence and prognosis of HFpEF. It is currently believed that depression may contribute to the pathogenesis of HFpEF through various interconnected mechanisms, including intestinal dysfunction, endothelial dysfunction, disturbances in fat and energy metabolism, autonomic nervous system dysfunction, hypothalamic–pituitary–adrenal (HPA) axis imbalance, and sex differences. Both conditions often share common comorbidities and genetic predispositions. Notably, inflammation is implicated in all these mechanisms and processes, playing a key role at different stages and facilitating the interaction between the two diseases during their onset and progression. However, the connections between depression and HFpEF have not yet been systematically and thoroughly elucidated. This review aims to comprehensively summarize the associations between depression and HFpEF, focusing particularly on elucidating their interplay in pathogenesis from an inflammation perspective, with special emphasis on the adverse effects of depression on the development of HFpEF. Additionally, we evaluate the management approaches for patients with comorbid depression and HFpEF, which include both pharmacological and nonpharmacological treatments, highlighting that personalized and integrated interventions may offer significant benefits. The review underscores the importance of increased attention and early identification of patients with comorbid depression and HFpEF and is expected to provide valuable insights and guidance for the clinical management of this significant patient population.

## 1. Introduction

Heart failure (HF) represents the terminal stage of various cardiovascular diseases (CVDs), and its prevalence increases significantly with age, posing a major global health challenge [1, 2]. HF with preserved ejection fraction (HFpEF), defined as left ventricular ejection fraction (LVEF) ≥ 50% with symptoms and/or signs of HF and objective evidence of cardiac structural and/or functional abnormalities consistent with elevated left ventricular (LV) filling pressures, is a highly heterogeneous group of diseases, distinct from HF with reduced ejection fraction (HFrEF) in both pathogenesis and clinical characteristics [3]. Various epidemiological studies indicate that HFpEF accounts for nearly 50% of all HF types, a proportion comparable to that of HFrEF [4, 5]. In recent years, a notable shift has been observed, with the incidence of HFpEF increasing while that of HFrEF has decreased [6]. HFpEF is more prevalent in women, and its prevalence increases more rapidly with age than that of HFrEF [7, 8]. Importantly, patients with HFpEF tend to exhibit poorer functional status and a higher prevalence of chronic comorbidities compared to those with HFrEF [9]. These comorbidities include underlying CVDs, obesity, diabetes, chronic kidney disease, chronic obstructive pulmonary disease (COPD), sleep apnea syndrome, and emotional disorders, among others [10, 11]. Some of the comorbidities are not only closely linked to the development of HFpEF but may also mediate its onset and progression to a certain extent.

Major depressive disorder (MDD), characterized by persistent sadness and loss of interest, has seen its global incidence surge from 172.7 million in 1990 to 274.8 million in 2019, a 59% increase that continues to escalate, with a consistently higher prevalence in women than in men. This has imposed a significant and growing disease burden due to the high levels of disability associated with depression [12, 13]. MDD is frequently comorbid with a range of other medical conditions, including CVDs, diabetes, metabolic syndrome, cancer, neurological diseases, osteoporosis, and chronic pain [14]. Currently, the epidemiological association between depression and HF is relatively well established. In Western countries, it has long been reported that over 30% of HF patients are affected by depressive disorders, and an even greater number exhibit depressive symptoms [15]. Multiple large‐scale, high‐quality clinical studies have shown that the severity of depressive symptoms significantly correlates with an increased risk of HF, establishing depression as an independent risk factor [16, 17].

In light of these findings, the 2026 European Society of Cardiology (ESC) Guidelines for the management of HF recommend that assessment of anxiety and depression should be considered in patients with HF to support personalized care plans and to identify factors that may contribute to adverse outcomes (Class IIa, Level C) [3]. Similarly, the American College of Cardiology (ACC), American Heart Association (AHA), and the Chinese Society of Cardiology (CSC) advocate routine screening for depression in HF patients when there is clinical suspicion [2, 18]. The majority of ongoing studies primarily focus on the correlation between depression and patients with HFrEF. However, depression has also been epidemiologically linked to the HFpEF population [19–22], which may be attributable to the complex interplay between depression and HFpEF in their shared pathophysiological mechanisms. Inflammatory responses are a common feature of both depression and HFpEF, serving as a crucial pathway for their mutual interaction [23]. Examining these conditions from this perspective may help elucidate the complex mechanistic links between them.

To date, no comprehensive summary has been provided regarding the connections between depression and HFpEF. This review comprehensively assesses the evidence from clinical and preclinical studies related to HFpEF and depression, focusing on the role of inflammation in the interplay of the pathogenesis of these two diseases, with particular attention to the potential mechanisms that may link depression to the progression of HFpEF. We also review the current potential therapeutic strategies for the comorbidity of HF and depression. This endeavor aims to provide insights that may facilitate the management of patients with these comorbidities.

## 2. Epidemiological Correlation Between HFpEF and Depression

Depression is a common comorbidity among patients with HFpEF [19]. Warraich et al. [21] assessed 202 elderly subjects (≥60 years) with HF and found that nearly 58% of individuals with HFpEF exhibited depressive symptoms, which were identified as significantly associated with HFpEF. Notably, only 16% of all participants had a documented clinical diagnosis of depression, indicating the frequent neglect of depressive symptoms in most HF patients [21]. In another study of 102,997 US patients admitted for HF in 2018,11% met the criteria for MDD. Among these, the prevalence of HFpEF was 13.9%, comparable to that of HFrEF (10%) [24]. Similarly, in a separate Japanese study involving 106 HF outpatients, the prevalence of depression and the Center for Epidemiologic Studies Depression (CES‐D) scale score did not significantly differ between HFrEF and HFpEF [25]. A cohort study by Vu et al. [20], involving 5086 patients with HF, demonstrated that HFpEF, but not HFrEF, was associated with a higher prevalence of depression compared to controls without HF. Intriguingly, over a 5.5‐year follow‐up, higher depression scale scores were associated with an increased risk of incident HFpEF but not HFrEF, independent of common comorbidities, cardiac structure and function, and prognostic biomarkers [20].

Compared with patients with HFrEF, depressive disorders or depressive symptoms seem more strongly associated with HFpEF and tend to be correlated with a worse prognosis. It has been reported that both outpatient and inpatient individuals with HFpEF exhibit higher depression scores compared to those with HFrEF and the non‐HF population [26, 27]. The presence of comorbid depression is associated with markedly diminished quality of life in HFpEF patients, and depressive symptoms are a more potent predictor of adverse outcomes in HFpEF compared to those in HFrEF [26, 28]. Several studies have further demonstrated that depression is associated with an increased risk of adverse cardiac events, cardiovascular mortality, and all‐cause mortality among patients with HFpEF [11, 25, 29]. Zhao et al. [30] categorized depression by severity and found that changes in depressive grade were more strongly associated with cardiovascular deaths in HFpEF patients rather than HFrEF patients after 1 year of follow‐up. Their focus on the prognostic significance of fluctuating depression levels is particularly noteworthy, suggesting a dynamic association between the course of HFpEF and depression [30]. Xing et al.’s [31] trajectory analysis of 609 HFpEF patients from the TOPCAT trial identified four depression patterns, including “recurrent deterioration” (high baseline scores, remission, then escalation) in 16.1% of participants, which was associated with significantly higher all‐cause mortality than the low trajectory, further underscoring this dynamic relationship. A prospective cohort study involving 26,268 participants free of HF from all 48 continental US states revealed that, among individuals without coronary artery disease (CAD) at baseline, depressive symptoms were independently associated with subsequent hospitalization for HFpEF but not for HFrEF or among those with pre‐existing CAD [22]. Consistent with these findings, a Japanese study revealed that depressive symptoms are an independent predictor of readmission rates for HFpEF but not for HFrEF [32]. Emerging evidence also supports a reverse direction: depression not only worsens HFpEF outcomes but may also arise secondary to HFpEF. In the PREDICT‑HFpEF trial, Lurz et al. [33] followed 202 elderly individuals with cardiovascular risk but no HF at baseline (median 5 years). Baseline depressive symptoms did not predict incident HFpEF; however, patients who developed HFpEF had significantly higher depression scores at follow‑up, which correlated with worse NYHA class, reduced daily activity, and lower quality of life [33].

The epidemiological association between depression and HFpEF is well established (Table 1). Most clinical findings are consistent with depression being associated with HFpEF progression, while some findings also suggest a potential reverse pathway, implying a possible bidirectional relationship. Mendelian randomization (MR) studies may offer additional insights into directionality. Large‑scale bidirectional MR analyses generally suggest a unidirectional causal effect of MDD on HF, without clear evidence for reverse causation (e.g., Liu et al. [35] reported a 16.9% increased risk [36]); however, other bidirectional MR studies have not supported a causal role of HF on depression [37–39]. Nevertheless, existing HF genome‑wide association study (GWAS) data do not distinguish HFpEF from HFrEF, a critical limitation, as recent MR evidence indicates non‑overlapping genetic targets between the two subtypes [40]. Therefore, these MR findings may not fully reflect the relationship specific to HFpEF. Moreover, some studies have failed to detect an association between depression and HFpEF incidence or prognosis, possibly due to insufficient adjustment for confounders (e.g., medications, psychotherapy, and social support) or inadequate follow‑up duration [24, 34]. Further high‐quality clinical studies and HFpEF‐specific genetic analyses are needed, and greater clinical attention should be paid to patients with comorbid conditions.

**Table 1:** Recent clinical studies on the link between depression and HFpEF.

Author	Year	Ethnic group of study	Numbers of study population	Main findings
Warraich et al. [21]	2018	American	HFpEF: 96 HFrEF: 106	Compared with HFrEF patients, HFpEF patients had more depressive symptoms by GDS score (5.4 ± 0.3 vs. 4.4 ± 0.4, *p* = 0.046).
Thyagaturu et al. [24]	2021	American	HFpEF: 36,594 (MDD patients were 13.9%) HFrEF: 66,403(MDD patients were 10%)	MDD was associated with similar combined 30‐day mortality and readmissions for both HFrEF and HFpEF.
Vu et al. [20]	2021	American	*N* = 5086	Depression scale score was associated with heightened risk of incident HFpEF (hazard ratio [HR] [95% CI], 1.06 [1.04–1.12]; *p* = 0.02), but not HFrEF (HR [95% CI], 1.02 [0.96–1.08]; *p* = 0.54).
Chandra et al. [11]	2020	American and Canada	*N* = 1413	HFpEF patients with higher baseline depressive symptoms and worsening depressive symptoms were associated with all‐cause mortality (hazard ratio [HR]:1.09; 95% confidence interval [CI]:1.02 to 1.16; *p* = 0.011).
Li et al. [29]	2022	China	*N* = 3456. (12.3% of patients had depression).	Postdischarge depression increased all‐cause mortality (HR 2.38; 95% CI: 1.93–2.94; *p* < 0.001) and composite events (HR 1.78; 95% CI: 1.55–2.05; *p* < 0.001) in acute HF patients. The mortality association was significantly stronger in HFpEF vs. HFrEF (*p* = 0.036).
Zhao et al. [30]	2024	China	HFpEF: 506 HFrEF: 439	In female patients, baseline depression class most strongly predicted cardiovascular death in HFrEF, whereas change in depression class showed a stronger prognostic trend in HFpEF.
Khodneva et al. [22]	2022	American	*N* = 26,268	In CHD‐free individuals, depressive symptoms predicted incident HFpEF hospitalization (HR 1.48; 95% CI: 1.00–2.18), but not HFrEF or those with baseline CHD.
Setoguchi et al. [32]	2015	Japan	HFpEF: 142 HFrEF: 168	Depression is an independent risk factor for readmission among patients with HFpEF (HR: 7.185).
Kato et al. [25]	2012	Japan	HFpEF: 61 HFrEF: 45	Depressive symptoms are common in HF patients and independently predict adverse events in HFrEF/HFpEF patients.
Liu et al. [34]	2021	China	*N* = 1431 (26.7% of patients had depression.	Baseline major depression did not increase mortality/rehospitalization in HFpEF; antidepressants might not improve prognosis.
Bekfani et al. [26]	2021	Germany	HFpEF: 17 HFrEF: 18 Non‐HF controls: 20	Patients with HFpEF showed worse QoL and higher anxiety and depression scores (6.5 [3.5–10.0] vs. 3.0 [1.0–6.5] vs. 2.0 [0.75–3.0], *p* = 0.01) compared with HFrEF and non‐HF controls.
Cha et al. [28]	2025	American	HFpEF: 426 HFrEF: 576	In both HF subtypes, depressive symptoms associated with highest risk. In HFrEF, depressive symptoms + physical inactivity predicted the combined endpoint; in HFpEF, depressive symptoms alone were the strongest predictor.
Shah et al. [19].	2013	270 sites in 6 countries	3445 patients with symptomatic HFpEF	TOPCAT cohort similar to contemporary HFpEF: low activity, reduced QoL, and depression common (27% at baseline).
Jonsson et al. [27].	2020	Sweden	HFpEF: 930 HFrEF: 3831	Mean EQ‐5D index unchanged at 1‑year in both groups. HFpEF: proportions with moderate/severe problems increased in all five dimensions. HFrEF: only three dimensions increased, with no change for anxiety/depression and improvement in usual activities.
Xing et al. [31]	2025	American	*N* = 609 (TOPCAT; HFpEF: 100%)	Four depression trajectories: low (57.3%), mild (18.1%), high (8.5%), and recurrent deterioration (16.1%). Recurrent deterioration trajectory increased all‐cause mortality (HR: 2.05; 95% CI: 1.16–3.64). No significant differences among low, mild, and high groups.
Lurz et al. [33]	2025	German	*N* = 202 (at‐risk without HF at baseline)	Prospective 5‐year follow‐up. Baseline HADS depression/anxiety did not predict incident HFpEF. HFpEF developers (15%) had higher follow‐up HADS depression scores (5.1 ± 3.5 vs. 3.5 ± 3.3, *p* = 0.01), which correlated with worse NYHA class, reduced activity, lower quality of life, and worse right ventricular function (all *p* < 0.05).

Abbreviations: CHD, coronary heart disease; EQ‐5D, EuroQol five dimensions questionnaire; GDS, geriatric depression score; HADS, Hospital Anxiety and Depression Scale; HF, heart failure; HFpEF, heart failure with preserved ejection fraction; HFrEF, heart failure with reduced ejection fraction; MDD, major depressive disorder; NYHA, New York Heart Association; QoL, quality of life; TOPCAT, trial of aldosterone blockade in patients with heart failure.

## 3. Evidence on the Association Between Depression and HF Progression

Evidence from human studies suggests that depression is associated with cardiac damage and the development of HF. A cross‐sectional study found that individuals with moderate‐to‐severe depression but no prior CVD had impaired LV diastolic function and increased LV mass index, which are pathological features of HFpEF [41]. Moreover, in non‐HF depressed populations, higher depression severity is associated with more pronounced LV remodeling and poorer diastolic indices [20]. Depression is associated with the exacerbation of multiple pathological processes including cardiomyocyte injury/necrosis, pathological hypertrophy, and interstitial fibrosis, which may impair cardiac structure and function. For example, studies have shown that chronic stress in rats is associated with significant myocardial hypertrophy and alterations in the myocardial extracellular matrix [42]. Similarly, chronic social defeat stress in mice or rats has been associated with electrocardiographic conduction disturbances and myocardial interstitial fibrosis [43, 44]. Conversely, there is some evidence that HFpEF itself may promote depressive symptoms. In a mouse model of HFpEF, researchers found that HFpEF, but not HFrEF, induced depression‐like behavior accompanied by increased IL‐33 expression in the amygdala [45]. Clinically, patients with HFpEF experience polypharmacy, frailty, and social isolation, each a well‑established depression risk factor [46].

While the association between depression and HFpEF has been consistently observed, it is important to recognize that most human studies cited above are observational in nature (cross‐sectional or prospective cohort studies) and cannot establish causality. The observed relationships could be influenced by reverse causation (e.g., subclinical cardiac dysfunction leading to depressive symptoms) or residual confounding from shared risk factors (e.g., physical inactivity). Animal studies provide supportive pathophysiological insights, but their translational relevance to human HFpEF requires further validation. Nevertheless, a genuine association between depression and HFpEF remains likely, given the consistency of the available evidence.

## 4. Inflammation in HFpEF and Depression

Emerging evidence highlights distinct pathophysiologies between HFpEF and HFrEF, with inflammation exerting a more prominent role in HFpEF [47, 48]. Elevated serum levels of proinflammatory cytokines are consistently observed in HFpEF cohorts [23]. Elevated tumor necrosis factor‐α (TNF‐α) and IL‐6 levels have been reported to specifically predict HFpEF incidence rather than HFrEF [48]. The inflammatory landscape in HFpEF is characterized by chronic low‐grade activation at both systemic and myocardial levels, primarily driven by inflammation mediated by innate and innate‐like cells, and is closely associated with comorbidities such as obesity, hypertension, aging, chronic kidney disease, and COPD, among others [47, 49]. Experimental studies show that IL‐1β knockdown reduces cardiac fibrosis and transforming growth factor‐β1 (TGF‐β1) expression in HFpEF murine models [50]. Similarly, inhibiting neutrophil extracellular traps (NETs) in HFpEF mice decreases cardiac macrophage infiltration and inflammation, improving cardiac fibrosis and diastolic function [51].

Recent studies have shown a bidirectional link between depression and inflammation [52]. Clinical studies have shown that depressive disorders are linked to heightened systemic inflammation, marked by elevated levels of C‐reactive protein (CRP) and proinflammatory cytokines in patients with MDD [53, 54]. Similarly, animal models of depression reveal significant increases in peripheral proinflammatory mediators [55]. This proinflammatory state may stem from multifactorial sources, including psychosocial stress, obesity, smoking, altered gut permeability, allergies, and disrupted circadian rhythms [56]. Conversely, inflammatory responses may worsen neuropsychiatric dysfunction through sustained proinflammatory cytokine release, microglial activation, increased blood–brain barrier (BBB) permeability, and disrupted neurotransmitter metabolism, particularly in the serotonin and dopamine systems. These mechanisms collectively impair cerebrovascular integrity and neural circuit function, promoting depressive symptoms [57]. This topic has been well reviewed elsewhere [58, 59]. Recent studies also highlight the role of genetic polymorphisms in inflammation‐related genes and sex‐specific neuroimmune interactions in shaping depression vulnerability [60, 61].

Inflammation is a crucial link between depression and the pathogenesis of HFpEF. After accounting for inflammatory markers, the correlation between depression and adverse cardiovascular events, as well as mortality, was reduced by 20% and 12.7%, respectively [62, 63]. However, elucidating how inflammatory pathways mediate the interplay between these two conditions remains a critical scientific question.

## 5. Mechanisms of Interaction Between Depression and HFpEF Based on Inflammatory Responses

### 5.1. Dysregulation of Intestinal Function

Gut‐derived inflammation serves as a crucial link in the pathogenesis of depression and HFpEF given its key role in gut‐heart and gut–brain communication [64, 65]. Altered intestinal permeability is a key contributor to gut‐derived systemic inflammation in HF. HF‐induced intestinal ischemia disrupts the intestinal epithelial barrier, increasing permeability and enabling bacterial metabolites and endotoxins to enter circulation, thus triggering systemic inflammation. This condition is commonly referred to as “leaky gut syndrome” [66]. The expression of intestinal epithelial tight junction proteins, such as occludin and claudin‐1, is reportedly diminished in both HFpEF and HFrEF [67, 68]. Depression‐related over‐activation of the hypothalamic–pituitary–adrenal (HPA) axis, abnormal enteric nervous activity, BBB disruption, neurogenic mediator release, and immune dysregulation can increase intestinal permeability and systemic inflammation, as evidenced by elevated levels of circulating inflammatory mediators, such as lipopolysaccharides (LPSs), Toll‐like receptor 4 (TLR4), nuclear factor kappa‐B (NF‐κB), and NOD‐like receptor pyrin domain‐containing 3 (NLRP3) [69, 70]. The release of enterogenic inflammatory mediators in depression promotes vascular and myocardial inflammation, thereby facilitating the development of HFpEF [71].

Alterations in the composition of the intestinal microbiota and shifts in their metabolites may represent another critical mechanism linking depression and the development of HFpEF, wherein inflammatory and metabolic pathways likely serve as pivotal mediators [72]. An elevated abundance of proinflammatory bacteria (e.g., Enterobacteriaceae and Desulfovibrio) and a reduced abundance of short‐chain fatty acid (SCFA)‐producing bacteria (e.g., Faecalibacterium) are hallmark features of the gut microbiota composition in individuals with depression [73, 74]. Reduced SCFA production results in diminished energy supply to gut cells, decreased mucin production, and compromised tight junction integrity, thereby disrupting normal intestinal barrier function and motility regulation and driving systemic inflammation and cardiac damage [75]. Trimethylamine N‐oxide (TMAO), another gastrointestinal metabolic byproduct that is upregulated in depression, is also associated with the risk of HFpEF [76, 77]. TMAO can directly promote myocardial hypertrophy and fibrosis and exacerbate myocardial inflammation by increasing TNF‐α levels and reducing IL‐10. TMAO can also induce vascular inflammation and damage the heart by activating multiple inflammation‐related signaling pathways, such as NF‐κB, the SIRT3‐SOD2‐mtROS axis, and the AMPK‐SIRT1 pathway [78–80].

In summary, there appears to be crosstalk between the brain–gut–heart axis during the pathogenesis of depression and HFpEF, with gut‐derived inflammatory activation as a pivotal factor (Figure 1). Targeting this intermediary process may hold therapeutic potential for comorbid depression and HF, although further experimental evidence is warranted.

**Figure 1:**
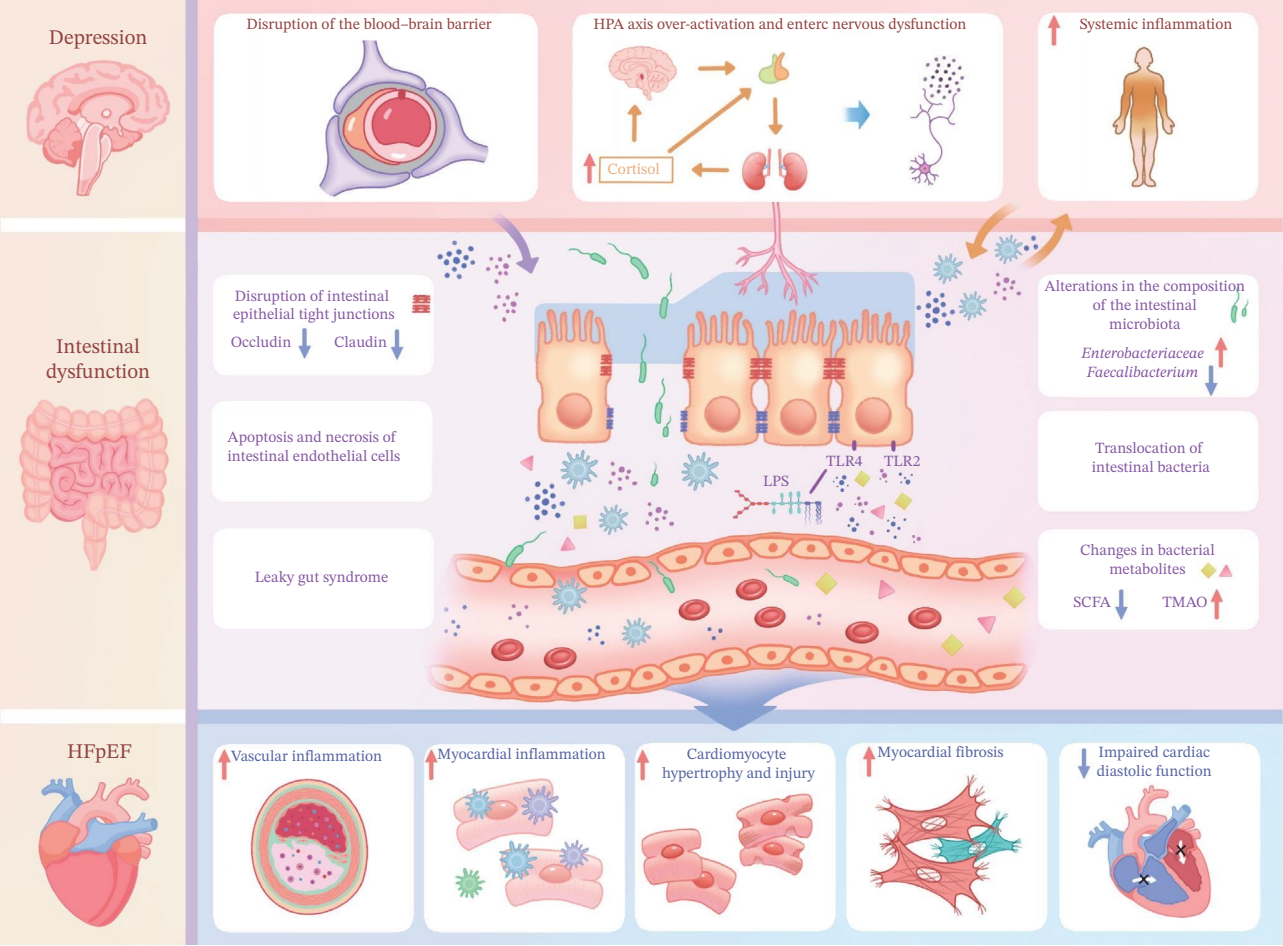
Depression promotes the progression of HFpEF via the brain–gut–heart axis, with inflammation playing a key role. Depression can influence gut function through various pathways, including disruption of the blood–brain barrier, abnormal activation of the HPA axis, enteric neural disorder, and systemic inflammation activation. On one hand, it increases gut permeability by mediating apoptosis and necrosis of gut endothelial cells and disrupting tight junctions, which leads to the release of gut‐derived proinflammatory mediators (such as proinflammatory cytokines and LPS). On the other hand, it exacerbates gut‐derived inflammation by altering the composition and structure of the gut microbiota, promoting bacterial translocation, and changing bacterial metabolites (e.g., reducing SCFA production and increasing TMAO production). These alterations upregulate systemic inflammatory responses and activate vascular and cardiac inflammation, which in turn mediate pathological processes such as cardiomyocyte hypertrophy, injury, necrosis, apoptosis, and cardiac interstitial fibrosis. Ultimately, these changes promote cardiac remodeling and the development of HFpEF.

### 5.2. Endothelial Dysfunction

Endothelial dysfunction and microvascular alterations are common in both depression and HFpEF [81, 82], with inflammation being a crucial driver [83]. Endothelial dysfunction under inflammatory conditions promotes HFpEF via multiple mechanisms, such as nitric oxide (NO) dysregulation and endothelial cell secretory dysfunction [84], which are also closely related to depression and exert bidirectional influences [85–87]. Depression may exert detrimental effects on cardiovascular endothelial function by increasing systemic chronic inflammation levels. Considerable evidence suggests that individuals with depression or depressive symptoms, whether past or current, are more susceptible to endothelial dysfunction, which is often accompanied by upregulated expression of proinflammatory cytokines, chemokines, vascular adhesion molecules, and decreased vascular anticoagulant activity [83, 88]. Importantly, systemic endothelial dysfunction in depression alters BBB permeability [89, 90]. This enables brain‐derived antigens and extracellular vesicles from damaged brain cells to enter the circulation, interact with peripheral immune cells, and trigger inflammatory responses that affect the heart [91]. Moreover, BBB dysfunction can induce autonomic dysregulation, which may in turn indirectly impair cardiovascular function [92, 93]. Consistently, imaging markers of neuroinflammation have been found to be significantly correlated with cardiac function [94]. Although neuroinflammation in depression may be relatively mild compared to that in acute brain injuries such as stroke, increased BBB permeability is still supposed to play a significant role in facilitating the entry of proinflammatory signals from the brain into the systemic circulation, thereby promoting myocardial inflammation and the progression of HFpEF to some extent [91].

### 5.3. Dysregulation of Fat and Energy Metabolism

#### 5.3.1. Obesity and Lipid Metabolism Disorders

Depression often co‐occurs with obesity, and more severe depression is associated with higher body mass index (BMI) and increases the risk of developing obesity over time [95]. This is primarily attributed to behavioral changes, such as increased emotional eating and reduced physical activity, as well as metabolic disorders [96, 97]. Overweight and obesity are also highly prevalent among HFpEF patients, with a rate of 83%. Obese individuals have a higher risk of developing HFpEF compared to HFrEF and often exhibit distinct hemodynamic and cardiac structural abnormalities [23, 98, 99]. Relevant evidence indicates that obesity and lipid metabolism disorders, as shared risk factors, may mediate the link between depression and HFpEF, closely tied to inflammation activation (Figure 2).

**Figure 2:**
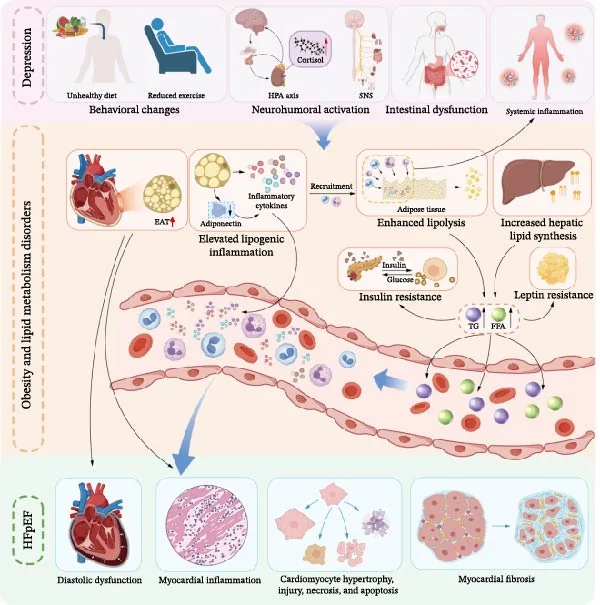
Obesity and lipid metabolism disorders in depression promote the progression of HFpEF through inflammation activation. In a state of depression, changes in lifestyle habits, such as increased emotional eating and decreased physical activity, overactivation of the HPA axis and the sympathetic nervous system, elevated systemic inflammation levels, and gastrointestinal dysfunction can lead to obesity and lipid metabolism disorders, which in turn damage the heart. Specifically, an increase in epicardial adipose tissue (EAT) can directly impair cardiac filling and indirectly cause myocardial injury and remodeling by inducing myocardial inflammation and metabolic alterations. Adipose tissue and adipocytes release more proinflammatory mediators, and further recruit inflammatory cells and amplify the inflammatory cascade. Additionally, increased lipolysis of adipose tissue and enhanced hepatic lipid synthesis can elevate circulating free fatty acids (FFAs) and triglyceride (TG) levels, aggravating insulin resistance and leptin resistance, further exacerbating lipid disorders, activating inflammatory signaling, and intensifying inflammation. Collectively, these interrelated processes promote cardiomyocyte hypertrophy, injury, and cardiac fibrosis, thereby facilitating cardiac remodeling and the development of HFpEF.

Depression may induce cardiac damage via lipogenesis‐associated inflammatory responses. Research indicates that persistent nutrient overload in depression can saturate adipocytes’ capacity to regulate fat metabolism and synthesis, triggering adverse effects such as leptin resistance, decreased adiponectin secretion, and increased proinflammatory factor release [100, 101]. Additionally, depression‐induced HPA axis over‐activation (e.g., elevated cortisol), gastrointestinal dysfunction (e.g., reduced SCFA and abnormal bile acid metabolism), inflammation‐driven insulin resistance, and sympathetic nervous system (SNS) excitation can all contribute to lipid metabolism abnormalities, including increased hepatic lipid synthesis and enhanced adipose tissue lipolysis, thereby exacerbating systemic and cardiovascular inflammation [100, 102–104]. Under inflammatory stimulation, adipocytes secrete mediators that recruit monocytes into adipose tissue, amplifying the inflammatory cascade and exacerbating systemic and cardiovascular inflammation [105].

Depression‐related lipid abnormalities may induce myocardial inflammation and injury by directly modulating cardiomyocyte metabolism. Patients with HF and comorbid depression exhibited elevated serum free fatty acids (FFAs) [106], linked to adverse outcomes in HF, including HFpEF [107]. Elevated FFA levels upregulate fatty acid oxidation genes in cardiomyocytes, enhancing FFA metabolism, causing intracellular triglyceride (TG) accumulation, increased reactive oxygen species (ROS), and inflammation, leading to cardiomyocyte hypertrophy, apoptosis, and dysfunction [105, 108]. It has recently been reported that modulating adiponectin receptor signaling can restore myocardial fatty acid balance, reduce cardiac lipid accumulation and fibrosis, and alleviate HFpEF [109]. Moreover, myeloid maladaptive fatty acid metabolism also plays a significant role in promoting systemic inflammation and HFpEF [110].

Moreover, individuals with depression have been reported to exhibit increased epicardial adipose tissue (EAT), which correlates positively with the severity of depression [111, 112]. EAT can induce myocardial inflammation and metabolic alterations, contributing to myocardial injury and remodeling. It also impairs cardiac function by restricting heart performance, as evidenced by increased cardiac filling pressures and elevated LV eccentricity [113]. The EAT may be more prominently implicated in the depression‐associated impairment of cardiac function in HFpEF.

#### 5.3.2. Mitochondrial Energy Dysfunction

Myocardial mitochondrial dysfunction is commonly observed in HFpEF [114]. Patients with depression also exhibit systemic mitochondrial dysfunction, characterized by abnormal expression of related biomarkers, including fibroblast growth factor 21 (FGF21) and growth differentiation factor 15 (GDF15), as well as perturbations in sphingolipid and phospholipid metabolism, elevated markers of mRNA and tRNA turnover, and disruptions in purine metabolism and purinergic signaling pathways [115, 116]. Inflammatory activation is a significant inducer of mitochondrial dysfunction in the brain, heart, and systemic circulation [117, 118]. Recent studies show that LPS administration in mice triggers a strong immune response, increasing mitochondrial superoxide production, reducing ATP synthesis, and decreasing mitochondrial membrane potential in the hippocampus [119]. Overproduction of IL‐1β and IL‐18, along with enhanced NLRP3 inflammasome assembly on hyperacetylated mitochondria, contributes to myocardial fibrosis in HFpEF murine models [120]. A recent study identified circulating mitochondria (C‐mito) as a novel mediator of interorgan communication, significantly influencing the heart–brain interaction. Specifically, C‐mito from HF mice induce endothelial cell‐derived neuroinflammation in the subfornical organ (SFO), a key cardiovascular autonomic center, thereby exacerbating myocardial sympathetic hyperactivation and pathological remodeling [121]. SFO neurons have recently been reported to modulate inflammation‐induced psychiatric disorders, such as anxiety and depression [122]. Therefore, mitochondrial dysfunction may play a role in the association between the pathological processes of depression and HFpEF, while inflammation acts in a mutually reinforcing way during these processes.

### 5.4. Autonomic Nervous System Dysfunction

Autonomic dysfunction in depression is primarily characterized by sympathetic overactivation and vagal inhibition [123]. The pattern of autonomic imbalance in HF patients mirrors that in depression and impacts cardiac function, structure, and prognosis via multiple pathways, such as immune‐inflammatory dysregulation, impaired baroreceptor reflex sensitivity, and reduced heart rate reserve [124].

Depression‐induced autonomic dysfunction disrupts the neuro‐immune axis, causing dysregulation of immune control and increasing systemic inflammation, thereby contributing to cardiac remodeling and HFpEF. Specifically, SNS activation promotes myocardial inflammation by mobilizing immune cells [125] and directly activating β and α1 receptors on cardiomyocytes, leading to intracellular inflammation and upregulation of caspase‐1, NLRP3, and IL‐18, which contribute to cardiomyocyte injury [126, 127]. A deficiency in β2‐adrenergic receptors reduces myocardial inflammatory cell infiltration and mitigates injury [128]. Sympathetic dopaminergic neurons also exert proinflammatory effects by regulating hematopoietic stem cell behavior via D2 receptor‐mediated c‐Kit/ERK signaling [129]. The anti‐inflammatory effects of the parasympathetic nervous system are also crucial for regulating systemic inflammation levels [130]. The effects are mainly driven by acetylcholine targeting the α7 nicotinic acetylcholine receptor (α7nAChR) on immune cells [131]. The involved pathways, such as JAK/STAT/ERK, NF‐κB, and PI3K/AKT, are intricately linked to HF [132]. Recent studies have mapped heart–brain–immune circuits [133, 134], showing that cardiac signals reach the brain via vagal afferents and that brain outputs modulate peripheral immunity. In depression, sympathetic overactivation and vagal withdrawal may hijack this loop, fueling maladaptive inflammation in HFpEF.

### 5.5. HPA Axis Dysregulation

Hyperactivation of the HPA axis is prevalent in depression and is often accompanied by elevated systemic inflammation, potentially driving the pathophysiology of comorbid HFpEF and depression [135]. Elevated cortisol concentrations can directly influence myocardial inflammation, hypertrophy, and fibrosis through the activation of glucocorticoid (GR) and mineralocorticoid (MR) receptors in the cardiovascular system. It can also increase the risk of major HFpEF predisposing factors, including obesity, diabetes, and hypertension [136, 137]. HPA axis dysregulation also contributes to inflammation by increasing the susceptibility to both endogenous and exogenous infections. Endogenous infections mainly originate in the gut. Elevated corticotropin‐releasing hormone (CRH) can act on intestinal epithelial cells to increase gut permeability, promoting microbial translocation, which in turn activates the immune system and drives inflammation [138]. Impaired adaptive immunity due to HPA axis dysfunction induces exogenous infections, which in turn heightens the body’s susceptibility to external pathogens [55]. Thus, HPA axis dysregulation in depression may drive HFpEF progression via inflammation, yet its precise dynamics require further clarification.

### 5.6. Genetic and Epigenetic Determinants of Inflammatory Predisposition

Depression and HFpEF are highly polygenic and share certain overlapping genetic factors related to inflammation. Specific single nucleotide polymorphisms (SNPs) in the IL‐6 receptor (IL‐6R) gene can alter systemic inflammation by affecting the balance between IL‐6R signaling pathways and are linked to both HFpEF and depression [139, 140]. For example, the IL‐6 genotype rs1800795 significantly increases the risk of both diseases [141, 142]. The TNF‐α genotypes rs1799964 and rs1800629 have also been shown to negatively influence the prognosis of patients with comorbid depression and HF [141, 143, 144]. Epigenetic factors, such as microRNA (miRNA) regulation, may also influence the susceptibility of patients with comorbid conditions. For instance, miR‐155 and miR‐146a are abnormally expressed in patients with depression and can adversely affect the heart through inflammation‐related pathways [145–147]. Dysregulation of miR‐129–5 p is associated with both depression and HFpEF [148, 149]. MiR‐21, a significant contributor to cardiac fibrosis, inflammation, and hypertrophy, has recently been identified as an important independent predictor of HFpEF and is also linked to depression through its aberrant expression in oligodendrocytes [150, 151].

### 5.7. Sex Differences: A Critical Intersection in the Inflammatory Crosstalk

The preceding sections have delineated the key inflammatory pathways linking depression to HFpEF. Notably, nearly all these pathways are modulated by sex hormones, particularly estrogen. Both HFpEF and depression exhibit striking sex disparities: HFpEF has a skewed prevalence toward females, with women having a nearly double lifetime risk of developing HFpEF compared to HFrEF [152], while depression affects women at more than twice the rate of men [153]. Importantly, HFpEF in aging women and depression in menopausal women share a common biological foundation—the modulatory effect of estrogen on inflammatory responses [154].

Menopause‐related estrogen decline triggers a pro‑inflammatory shift across multiple cardiovascular compartments. At the microvascular level, estrogen loss impairs endothelial function through increased oxidative stress and reduced NO bioavailability, contributing to coronary microvascular dysfunction, a core pathological feature of HFpEF [155]. In the adipose tissue, estrogen deficiency exacerbates visceral fat accumulation and adipose tissue macrophage infiltration, leading to a depot‑specific inflammatory phenotype more pronounced in women [156]. In the gut, estrogen deficiency disrupts barrier integrity via the “estrogen‑gut‑vascular axis,” leading to microbial metabolite imbalances that drive vascular inflammation [157]. This adipose‑driven and gut‑derived inflammation is particularly relevant, as HFpEF in aging women exhibits a distinct “obesity, chronic inflammation, and microvascular dysfunction” phenotypic profile [154]. Importantly, the female heart itself shows a pro‑inflammatory shift with aging not seen in male hearts, and mood symptoms in HF patients are associated with inflammation among females only [158, 159].

Estrogen also maintains central immune homeostasis, influencing depression susceptibility. Menopause‑related estrogen decline presents a 2–5‐fold higher risk of developing depression [153]. Preclinical evidence from ovariectomized (OVX) mouse models shows that estrogen deficiency suppresses estrogen receptor β (ERβ) expression in the hippocampus and prefrontal cortex, coinciding with microglial‑derived neuroinflammation and decreased brain‐derived neurotrophic factor (BDNF) activity [160]. Estrogen replacement alleviates depressive‑like behaviors by inhibiting prefrontal proinflammatory cytokines [161]. The kynurenine pathway is also involved. Inflammatory stimuli upregulate indoleamine 2,3‑dioxygenase (IDO), shifting tryptophan metabolism toward neurotoxic kynurenine metabolites, a process modulated by estrogen. Notably, even healthy women display a baseline kynurenine pathway profile that resembles that of depressed patients, suggesting an intrinsic vulnerability. This vulnerability may remain latent under normal estrogen levels but becomes unmasked when estrogen levels declines [162].

The above observations can be integrated into a “two‑hit” inflammatory model in the context of estrogen decline. The first hit is the heightened systemic and cardiovascular inflammation that predisposes to HFpEF, and the second hit is the amplified neuroinflammation that promotes depression. These two hits interact bidirectionally. On the one hand, peripheral inflammatory cytokines cross the BBB, and sex‑specific differences in this process may explain women’s higher susceptibility to severe depression [163]. On the other hand, depression‑driven sympathetic activation and HPA axis dysregulation feedback to amplify systemic inflammation and accelerate HFpEF progression. This bidirectional interplay sustains the comorbid state.

In summary, menopausal estrogen decline unmasks aging women’s vulnerability to systemic and neuroinflammation. Their bidirectional interplay creates a self‑perpetuating cycle that sustains the comorbid state, highlighting the need for sex‑specific strategies to manage female patients with both depression and HFpEF.

## 6. Management of HFpEF With Comorbid Depression: From Recognition to Treatment

### 6.1. Screening and Differential Diagnosis

#### 6.1.1. Screening for Depression in HFpEF

Currently, the identification of patients with HFpEF and comorbid depression remains relatively insufficient. The 2026 ESC Guidelines for the management of HF recommend that assessment of anxiety and depression should be considered in patients with HF to support the development of personalized care plans and to identify factors that may contribute to adverse outcomes (Class IIa, Level C) [3]. This recommendation is consistent with prior guidance from the ACC/AHA and CSC, which advocate routine screening of HF patients with a validated questionnaire when depression is clinically suspected [2, 18]. Relevant scales include the Beck Depression Inventory, Cardiac Depression Scale, Hamilton Depression Scale, Patient Health Questionnaire‐9 (PHQ‐9), Geriatric Depression Scale, and Hospital Anxiety and Depression Scale, among others [2, 3, 15]. However, consensus on the optimal treatment for HF patients with depression has yet to be achieved. Moreover, screening instruments alone cannot distinguish true major depression from other forms of psychological distress that commonly arise in the context of chronic cardiac illness.

#### 6.1.2. Distinguishing Major Depression From Demoralization

A critical but often overlooked distinction in cardiology settings is that between major depression and demoralization. Demoralization is a syndrome of existential distress rooted in consultation‐liaison psychiatry, formally recognized in ICD‐11 as MB22.2, and well established in psycho‐oncology [164]. It is characterized by subjective incompetence, helplessness, hopelessness, loss of meaning and purpose, a sense of failure, and a diminished capacity to cope with a stressful life situation [165–167]. Although demoralization shares distress‐related symptoms with depression, it is distinguished from major depression by its core feature of subjective incompetence, whereas anhedonia is central to depression [165].

Demoralization is common and clinically consequential in cardiac patients. In chronic HF, demoralization syndrome has been identified in 57.7% of cases [168]. Among patients with supraventricular tachyarrhythmias, 23.6% showed high levels of demoralization, and 50% of these patients received no formal psychiatric diagnosis [169]. Recognition is important because demoralization responds preferentially to psychotherapeutic support aimed at restoring morale, meaning, and purpose rather than to antidepressant medication [165]. In acute coronary syndrome, a randomized controlled trial showed that cognitive behavioral and well‐being therapies significantly reduced demoralization compared with clinical management [170]. A systematic review of demoralization interventions in chronic diseases found meaning‐centered psychotherapy and dignity therapy to have the strongest empirical support, with most studies showing significant improvement [171]. Recognizing demoralization can therefore prevent inappropriate antidepressant prescribing, which exposes complex cardiac patients to proarrhythmic risks and drug interactions. These considerations underscore the need for careful psychiatric evaluation beyond symptom checklists to differentiate demoralization from major depression in patients with HFpEF and comorbid psychological distress.

### 6.2. Potential Therapeutic Approaches

#### 6.2.1. Nonpharmacological Therapies

##### 6.2.1.1. Physical Activity

Moderate physical exercise can improve the psychological state and prognosis of HF patients. Multidisciplinary physical rehabilitation interventions, comprising strength, balance, mobility, and endurance training along with home‐based exercises, provide more substantial benefits for HFpEF patients than those with HFrEF [172]. It has been shown that even mild exercise is associated with a reduction in depressive symptoms in HF patients [173]. The HF‐ACTION Randomized Trial showed that exercise training (ET) resulted in a modest reduction in depressive symptoms compared with guideline‐based usual care per week [174]. The HEART Camp trial demonstrated that ET intervention significantly alleviated depressive symptoms in HFpEF patients [175]. A 12‐week Tai‐chi regimen, an ancient Chinese martial art, improved mood and cardiorespiratory fitness in HFpEF patients, showing its potential as a safe and effective therapy for those with comorbid depression [176]. Sex differences in response to ET have also been observed in HFpEF. Women have lower baseline exercise capacity and quality of life than men, linked to postmenopausal inflammation and microvascular dysfunction [177]. However, a pooled analysis of 498 patients with HFpEF found that ET significantly improved percent‐predicted peak VO_2_ in women but not in men over 12 months [178]. Importantly, women adhere to cardiac rehabilitation as well as men and achieve similar gains in peak VO_2_ and quality of life [179]. Therefore, well‐timed and intensity‐adjusted exercise may help alleviate depressive symptoms in HFpEF patients.

##### 6.2.1.2. Cognitive Behavioral Therapy (CBT)

CBT can alleviate depressive symptoms in HF patients by modulating pathological cognitive and behavioral processes. A systematic meta‐analysis revealed that CBT is potentially more effective than conventional treatment in alleviating depression in HF patients, with the benefits persisting at the 3‐month follow‐up [180]. CBT has been confirmed to be superior to usual care for major depression in patients with HF in a randomized clinical trial [181]. Recent studies have consistently shown that short‐term CBT (<3 months) has limited impact on self‐care, depression, or anxiety in HF patients, whereas long‐term CBT (4–9 months) significantly reduces anxiety and depression [182]. Further research is needed to elucidate the specific efficacy of CBT in HFpEF patients with depression.

##### 6.2.1.3. Vagus Nerve Stimulation (VNS) Therapy

The VNS therapy has demonstrated its unique role in the treatment of HFpEF and depression. Chronic intermittent low‐level tragus stimulation (LLTS), a type of low‐level transcutaneous VNS, has been reported to improve diastolic function, reduce cardiac inflammation and fibrosis, and increase survival in a rat model of HFpEF [183]. Consistently, a clinical randomized controlled trial showed that 3 months of LLTS neuromodulation significantly enhanced global longitudinal strain, reduced inflammatory cytokines, and improved quality of life in HFpEF patients [184]. An ongoing clinical study (ANTHEM‐HFpEF) is currently underway to evaluate the feasibility, tolerability, and safety of right cervical VNS in patients with HFpEF [185]. As early as 2005, VNS was approved by the US Food and Drug Administration (FDA) as a long‐term adjunctive treatment for patients over 18 years old with refractory MDD [186]. Thus, VNS may benefit patients with HFpEF and depression, although further evidence is needed.

#### 6.2.2. Pharmacological Therapy

##### 6.2.2.1. Antidepressants

The efficacy of antidepressants in the HF population has been preliminarily explored. Selective serotonin reuptake inhibitors (SSRIs) are the most widely used antidepressants and have a relatively favorable cardiovascular safety profile [187]. The SADHART‐CHF trial, a randomized, double‐blind, placebo‐controlled study of 469 patients with HF and MDD, demonstrated that sertraline was safe in patients with significant HF. However, sertraline did not significantly improve depressive symptoms compared with placebo (between‐group difference in Hamilton Depression Rating Scale change: −0.4; *p* = 0.89), nor did it improve cardiovascular outcomes [188]. These findings were corroborated by the MOOD‐HF trial, in which escitalopram also failed to show significant benefit over placebo in 372 patients with chronic systolic HF and depression [189]. Consistent with these findings, the 2026 ESC Guidelines for the management of HF state that the safety of sertraline and escitalopram has been demonstrated, although without a significant reduction in depression compared with placebo [3]. These considerations underscore the need for adequately powered, HFpEF‐specific trials to determine whether any antidepressant strategy can meaningfully improve depressive symptoms in this population.

Although the aforementioned randomized trials did not demonstrate a significant advantage of SSRIs over placebo for depressive symptoms, this does not exclude the possibility that SSRIs exert cardiovascular protective effects through mechanisms independent of mood improvement. For example, paroxetine has been shown to reduce cardiac remodeling and improve cardiac function in patients and animal models with hypertension or acute myocardial infarction by inhibiting G‐protein‐coupled receptor kinase 2 (GRK2) [190–192]. In addition to GRK2 inhibition, SSRIs may improve cardiovascular status partly through their anti‐inflammatory effects. Sertraline and escitalopram can reduce circulating inflammatory mediator levels in patients with depression [88, 193]. Fluoxetine has been reported to modulate proinflammatory responses by upregulating IL‐10 expression, thereby mitigating the cardiac impact of sepsis [194]. Beyond classic SSRIs, vortioxetine, a multimodal antidepressant rather than a classic SSRI, was recently shown to markedly reduce CRP (by 89%) and TNF‐α (by 84%) and improve quality of life in patients with chronic HF and comorbid depression, providing direct evidence for dual anti‑inflammatory and antidepressant benefits [195]. Other serotonergic antidepressants, such as 5‐hydroxytryptamine 1A (5‐HT1A) receptor agonists (e.g., tandospirone), offer high cardiovascular safety and no risk of drug dependence, and have been proven to reduce cardiovascular events and improve prognosis [196, 197]. A review concluded that females generally respond better to serotonergic antidepressants than males, though the evidence remains inconclusive [198]. Mechanistically, estrogen modulates serotonergic signaling and drug metabolism and influences the kynurenine pathway [199, 200]. Whether these mechanistic benefits translate into clinical improvement in HFpEF remains to be determined.

Finally, tricyclic antidepressants (TCAs) are formally contraindicated in patients with HF. The 2026 ESC Guidelines explicitly recommend against their use because of a high proarrhythmic risk and the potential to cause hypotension, worsening HF, and arrhythmias [3]. This concern is particularly relevant in HFpEF, where atrial fibrillation is common and arrhythmic susceptibility is increased. TCAs should therefore be avoided in all patients with HF, and safer alternatives such as SSRIs or newer antidepressants should be preferentially considered.

##### 6.2.2.2. Anti‐Inflammatory Therapy

Emerging evidence suggests that therapies targeting inflammatory pathways may benefit HFpEF patients. A randomized controlled trial demonstrated that 14‐day treatment with anakinra, an IL1β receptor antagonist, markedly diminished the systemic inflammatory response and enhanced the aerobic exercise capacity in patients with HFpEF [201]. In a subsequent phase II clinical trial, anakinra administration for 12 weeks in patients with stable HFpEF and elevated CRP tended to reduce NT‐proBNP and hsCRP levels yet failed to improve peak oxygen consumption or ventilatory efficiency [202]. The CANTOS study demonstrated that canakinumab, an IL‐1β‐targeting monoclonal antibody, significantly reduced recurrent cardiovascular events, HF hospitalizations, and HF‐related mortality in patients with prior myocardial infarction and elevated CRP. The study population primarily consisted of older individuals with comorbidities such as obesity, diabetes, and hypertension, which are typical of the HFpEF phenotype [203, 204]. The SATELLITE trial is currently assessing the efficacy of AZD4831, a myeloperoxidase inhibitor, in HFpEF. While its safety is confirmed, its therapeutic potential for HFpEF remains to be fully elucidated due to early trial termination [205]. The use of anti‐inflammatory agents, such as nonsteroidal anti‐inflammatory drugs (NSAIDs), omega‐3 fatty acids, cytokine inhibitors, statins, and corticosteroids, in treating depression is an emerging research area. These agents show significant antidepressant efficacy in patients with MDD and are generally well tolerated, though their potential side effects and overall impact require ongoing evaluation [206, 207].

Given the potential benefits of anti‐inflammatory interventions in both HFpEF and depression, their application in comorbid patients appears promising. However, the 2026 ESC Guidelines do not currently recommend anti‐inflammatory therapies, including IL‐1β blockade, for the routine management of HFpEF, and these agents remain investigational [3]. Further research is needed to elucidate the underlying mechanisms, identify precise targets, and conduct high‐quality trials.

##### 6.2.2.3. Sodium‑Glucose Transport Protein 2 (SGLT2) Inhibitors

Beyond conventional anti‑inflammatory agents, several other drugs also possess anti‑inflammatory properties that may benefit this comorbid population. In the 2026 ESC Guidelines, SGLT2 inhibitors, together with mineralocorticoid receptor antagonists (MRAs), are recommended as part of foundational medical therapy for patients with symptomatic HF across the entire LVEF spectrum, including HFpEF, to reduce the risk of HF hospitalization or cardiovascular death (Class I, Level A) [3]. The SGLT2 inhibitors dapagliflozin and empagliflozin reduce the composite endpoint of HF hospitalization or cardiovascular death in symptomatic HFpEF, based on the EMPEROR‐Preserved and DELIVER trials [208, 209]. Their anti‑inflammatory properties provide a mechanistic link between their cardioprotective effects in HFpEF and potential mood‑modulating benefits in depression. SGLT2 inhibition suppresses NLRP3 inflammasome activation, a central inflammatory hub in both HFpEF pathophysiology and depressive disorders, through elevation of ketone bodies such as β‑hydroxybutyrate and reduction of mitochondrial ROS [210, 211]. Furthermore, SGLT2 inhibitors reduce pro‑inflammatory cytokines IL‑1β, IL‑6, IL‑17 and MCP‑1, while promoting anti‑inflammatory M2 macrophage polarization [211]. Sex differences in SGLT2 inhibitor responses have been evaluated, with both empagliflozin (EMPEROR‑Preserved) and dapagliflozin (DELIVER) providing similar cardiovascular benefits in women and men with HFpEF and supporting their universal use across sexes [208, 209].

Emerging clinical evidence supports the antidepressant potential of SGLT2 inhibitors. A 2024 randomized controlled trial demonstrated that empagliflozin as an adjunctive therapy to citalopram significantly improved depression severity in patients with MDD [212]. A 2025 network meta‑analysis of 62 randomized controlled trials comprising 200,068 participants further established that high‑dose dapagliflozin (10 mg/day) significantly reduces the incidence of depression, particularly in patients with type 2 diabetes [213].

Collectively, SGLT2 inhibitors exert dual benefits on comorbid depression and HFpEF through overlapping anti‑inflammatory and neuroprotective mechanisms. However, evidence supporting their therapeutic efficacy specifically in patients with established comorbid depression and HFpEF remains indirect, as most data derive from diabetic populations or HF trial subgroups. Dedicated studies in this specific patient population are warranted.

##### 6.2.2.4. Probiotics

Probiotics are live microorganisms that confer health benefits on the host when administered in adequate amounts [214]. They are the most extensively studied microbial‐targeted therapies for depression. A recent comprehensive study identified 178 probiotic strains and subspecies that can alleviate depression, with the majority belonging to the Lactobacillus and Bifidobacterium genera [215]. The administration of these probiotics has been shown to effectively lower inflammation levels and enhance endothelial function, consequently producing an antidepressant effect [214, 215]. In a porcine model of HFpEF, probiotic supplementation has been shown to effectively reduce gut epithelial permeability, attenuate systemic inflammation, and inhibit cardiac structural remodeling [71]. Furthermore, prebiotics, which are specifically defined as substrates that can be selectively utilized by host microorganisms to confer health benefits, have been shown to exert cardioprotective effects by reducing circulating endotoxin levels in rats [216]. Thus, modulation of the gut–heart/brain inflammatory axis via symbiotic interventions, including probiotics, prebiotics, adjunctive supplements, precision antibiotic therapies, and fecal microbiota transplantation, may confer therapeutic benefits in patients with comorbid depression and HFpEF [217]. However, the 2026 ESC Guidelines do not currently recommend probiotics or other microbiota‐targeted therapies for the routine management of HFpEF, and their use remains investigational [3].

Additionally, nonpharmacological treatments, such as dietary management, sleep management, and other types of psychological interventions, as well as pharmacological therapies like traditional Chinese medicine, GLP‐1 receptor agonists (GLP‐1 RAs), and certain immune modulators, may also benefit patients with comorbid HFpEF and depression [218–224]. The main potential interventions are summarized in Figure 3. Exploration of precision therapies based on the shared pathogenesis of these two diseases, along with the targeted use of integrated treatment strategies that combine pharmacological and nonpharmacological interventions according to the specific clinical characteristics of patients, is expected to benefit this patient population.

**Figure 3:**
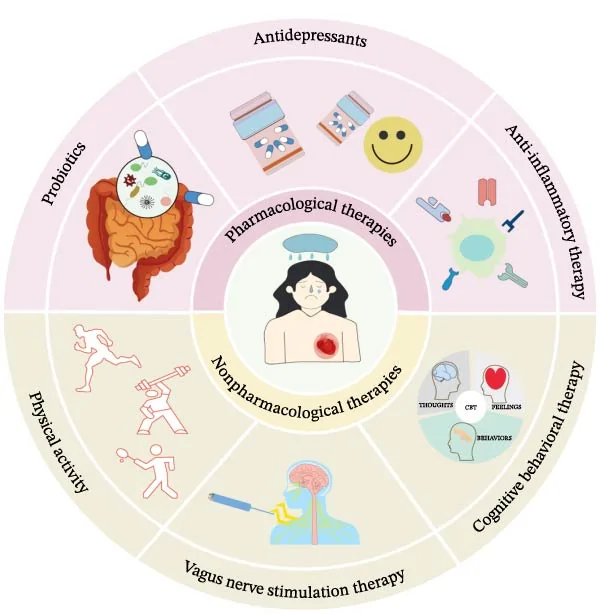
Potential interventions for HFpEF with comorbid depression. It is currently believed that certain interventions may benefit patients with depression comorbid with HFpEF. Nonpharmacological treatments include moderate physical exercise, cognitive behavioral therapy (CBT), and vagus nerve stimulation (VNS) therapy, among others. Pharmacological treatments encompass antidepressants, anti‐inflammatory therapy, and probiotics, among others.

## 7. Conclusion

Depression and HFpEF often coexist, typically resulting in a poorer quality of life and prognosis. Their mechanisms are thought to be bidirectionally linked, although most available evidence is observational and cannot establish causality. Depression is hypothesized to contribute to the pathological progression of HFpEF through multiple mechanisms. This article delineates the role of inflammation in these mechanisms, emphasizing its critical involvement in gut dysfunction, endothelial dysfunction, metabolic disorders, autonomic dysfunction, HPA axis dysfunction, and sex differences. It is worth noting that none of these mechanisms operate in complete isolation from the others. Centered on immune inflammation, the above mechanisms form a complex regulatory network, interacting with each other and collectively contributing to the progression of HFpEF under the state of depression. Future studies, including randomized controlled trials and in vivo/in vitro mechanistic investigations, are needed to directly test causal hypotheses, validate specific interactive pathways, and develop integrated interventions for this comorbidity.

## Author Contributions

**Hongfan Chen**: writing – original draft, writing – review and editing, visualization. **Nan Zhang**: writing – original draft, writing – review and editing, visualization. **Yufan Feng:** writing – review and editing. **Mengxing Tao:** writing – review and editing, supervision, funding acquisition. **Chi Geng:** conceptualization, writing – original draft, writing – review and editing, visualization, supervision, funding acquisition.

## Funding

This work was supported by the National Natural Science Foundation of China (Grant 82300438), the Jiangsu Provincial Double‐Innovation Doctor Program (Grant JSSCBS20230481), the Suzhou Gusu Medical Talent Program (Grant GSWS2023099), the Suzhou Science and Technology Research and Development Program (Grant SYWD2025136), and the Research Fund Project of the Second Affiliated Hospital of Soochow University (Grants SDFEYBS2212 and XKTJ‐XK202411).

## Conflicts of Interest

The authors declare no conflicts of interest.

## Data Availability

The data sharing is not applicable to this article as no datasets were generated or analyzed during the current study.
